# Presence of anti-nuclear antibody associated with worse clinical outcomes of anti-NMDAR encephalitis

**DOI:** 10.3389/fneur.2022.975583

**Published:** 2022-10-12

**Authors:** Chunyan Lei, Yongyu Li, Haijiang Li, Xiaoyan Zhu, Wen Jiang, Xiaolong Chang

**Affiliations:** Department of Neurology, First Affiliated Hospital of Kunming Medical University, Kunming, China

**Keywords:** anti-*N*-methyl-D-aspartate receptor encephalitis, clinical characteristic, prognosis, autoantibodies, anti-nuclear antibody

## Abstract

**Purpose:**

Systemic autoantibodies are important for the diagnosis of autoimmune diseases, but their roles in anti-*N*-methyl-D-aspartate receptor (anti-NMDAR) encephalitis are unknown. The purpose of our study is to investigate the characteristics and a prognosis of anti-NMDAR encephalitis with the prevalence of autoantibodies.

**Methods:**

Systemic autoantibodies were evaluated in 64 patients with anti-NMDAR encephalitis and 14 patients with autoimmune encephalitis with other forms. Then, according to systemic autoantibodies, patients with anti-NMDAR encephalitis were divided into an anti-nuclear antibody (ANA) positive group and an ANA negative group. The clinical outcome was assessed by a modified Rankin score at 12 months after the disease onset.

**Results:**

A total of 64 patients with anti-NMDAR encephalitis were enrolled, of which 28.13% (18/64) were positive for ANA. The titers of a positive anti-NMDAR antibody in CSF (*p* = 0.041) and serum (*p* = 0.031) in the ANA-positive group were significantly higher than the ANA-negative group. Patients with ANA positive than those with ANA negative showed lower rates of headache (*p* = 0.047) and speech disorder (*p* = 0.049). The presence of ANA was associated with a worse clinical outcome at 12 months (*p* = 0.043).

**Conclusion:**

ANA was prevalent in patients with anti-NMDAR encephalitis, and associated with a worse prognosis and impaired neurological recovery.

## Introduction

Anti-*N*-methyl-D-aspartate receptor (anti-NMDAR) encephalitis is a synaptic autoimmune disorder, which mostly affects young women and children (1, 2). Clinical manifestations mainly include psychiatric symptoms, impaired memory, and neurological symptoms like movement abnormalities, seizures, or autonomic instability (3, 4). Most patients are responsive to immunotherapies, which include intravenous immunoglobulins (IVIG), corticosteroids, or plasmapheresis (3, 4). Binding of IgG antibodies to NMDAR induces an invertible internalization of the receptors from extrasynaptic and synaptic spaces (5, 6). However, the accurate mechanisms of pathogenesis are not to be elaborated.

As known, positive autoantibodies may hint for ongoing autoimmune reactions, and measuring autoantibodies should be guided by clinical manifestations (7–10). Detection of autoantibodies is significant to diagnose many autoimmune diseases, such as systemic lupus erythematosus, autoimmune rheumatic diseases, systemic sclerosis, and idiopathic inflammatory myopathies (7–10). The rate of autoantibodies is highest in patients with systemic autoimmune rheumatic diseases. Moreover, these antibodies are also tested in patients with organ-specific autoimmune diseases, certain infections, advanced age, and in some healthy individuals. A timely diagnosis of anti-NMDAR encephalitis is a tremendous challenge due to the wide scope of many symptoms. The anti-NMDAR encephalitis is a disorder with complicated etiology. Thus, autoantibodies testing may be applied to evaluate the possibility of an anti-NMDAR encephalitis diagnosis, with related information coming from the identification of antibodies responding to certain intracellular targets (8, 9).

However, few studies have examined the effects of autoantibodies in anti-NMDAR encephalitis (7, 8). The purpose of our study is to examine autoantibodies in patients with anti-NMDAR encephalitis and assess potential associations between these autoantibodies and a prognosis and clinical features.

## Methods and materials

### Patients and evaluation

We retrospectively recruited 64 patients with anti-NMDAR encephalitis and 14 patients with autoimmune encephalitis with other forms from 01 January 2016 to 31 January 2020 at Department of Neurology, First Affiliated Hospital of Kunming Medical University. The study was approved by the First Affiliated Hospital of Kunming Medical University and conformed to the Declaration of Helsinki. All patients were screened for the presence of autoantibodies, including anti-NMDAR, anti-AMPAR, anti-LGI, anti-CASPR2, anti-GABAB, anti-DPPX, anti-DRD2, anti-GAD65 by the indirect immunofluorescence test of CSF/and serum samples.

### Baseline data collection

Demographic characteristics and clinical features of included patients were reviewed by two authors (YL and HL). Intensive care unit (ICU) admission and treatments were obtained. The results of laboratory tests (white blood cell count, protein concentration) and electroencephalography (EEG) also were collected.

The blood samples were obtained within 24 h after admission and immediately were tested. The autoantibodies included an anti-nuclear antibody (ANA), an anti-double-stranded DNA (dsDNA) antibody, an anti-chromosome antibody, an anti-ribosomal protein antibody, an anti-Sm antibody, an anti-SmRNP antibody, an anti-SSA antibody, an anti-SSA52 antibody, an anti-SSA60 antibody, an anti-SSB antibody, an anti-Scl-70 antibody, and an anti-centromere antibody based on the previous studies (7). Moreover, other antibodies also included an anti-neutrophil cytoplasmic antibody, an anti-myeloperoxidase antibody, an anti-protease 3 antibody, and an anti-glomerular basement membrane antibody. ANA, an anti-dsDNA antibody, an anti-neutrophil cytoplasmic antibody, and an anti-myeloperoxidase antibody were detected by the indirect immunofluorescence assay. The anti-protease 3 and anti-glomerular basement membrane antibody were detected by immunoblotting. The rest antibodies were detected by immunofluorescence.

### Clinical outcome

Functional outcomes were assessed at 12 months after the disease onset. The patients with an mRS score of 0 were regarded as completely recovered; mRS scores of 1–2 were mild deficit; mRS scores of 3–5 were severe deficit; mRS scores of 6 were dead. Poor clinical outcomes were classified as mRS scores of 3–6.

### Statistical analysis

The Statistical Package for the Social Sciences version 21.0 (SPSS, Chicago, IL, USA) was used for statistical analyses. Continuous data are described as mean [standard deviation (SD)] or/and median [interquartile range (IQR)], and categorical data are shown as counts (percentages). Student's *T*-test was used for intergroup comparisons of data with a normal distribution and homogeneous variance, while the Mann–Whitney *U* test was used for intergroup comparisons of data with a non-normal distribution and heterogeneous variance. Categorical variables were assessed using the Pearson's chi-squared or Fisher exact tests or the Fisher-Freeman-Halton test (an extension of the Fisher exact test for contingency tables larger than 2 × 2). The values of *p* < 0.05 were deemed to indicate statistically significant.

## Result

### Comparison of a clinical characteristic and autoantibodies between anti-NMDAR encephalitis and autoimmune encephalitis with other forms

Among the 78 patients, 64 patients (82.05%) were positive for an anti-NMDAR antibody; 8 patients (10.26%) for an anti-LGI1 antibody; 3 patients (3.85%) for an anti-GABABR antibody; 2 patients (2.56%) for an anti-AMPAR antibody, 1 patient (1.28%) for an anti-DPPX antibody. Table 1 shows the clinical characteristic and ANA in two groups. Patients with autoimmune encephalitis with other forms than those with anti-NMDAR encephalitis showed old age (40.86 ± 14.56 vs. 29.42 ± 17.43; *p* < 0.001), lower rates of fever (2 vs. 32; *p* = 0.018), headache (2 vs. 34; *p* = 0.016), and altered consciousness (2 vs. 38; *p* = 0.003), and a higher rate of seizures (10 vs. 26; *p* = 0.036). There was no statistically significant difference in gender, abnormal MRI, abnormal EEG, and ANA.

**Table 1:** The clinical characteristic between anti-NMDAR encephalitis and autoimmune encephalitis with other forms.

	**NMDAR encephalitis (*n* = 64)**	**Autoimmune encephalitis with other forms (*n* = 14)**	***P*-value**
Age, years (mean ± SD)	29.42 ± 17.43	40.86 ± 14.56	<0.001
Male (%)	36 (56.25)	10 (71.43)	0.296
Abnormal MRI (%)	34 (53.13)	8 (57.14)	0.785
Abnormal electroencephalography (%)	26 (40.63)	8 (57.14)	0.259
Fever	32 (50.00)	2 (14.29)	0.018
Headache	34 (53.13)	2 (14.29)	0.016
Dizziness	10 (15.63)	0 (0.00)	0.194
**Clinical symptoms (%)**			
Abnormal behavior	44 (68.75)	6 (42.86)	0.067
Speech disorder	8 (17.39)	0 (0.00)	0.338
Seizures	26 (40.63)	10 (71.43)	0.036
Memory disorder	6 (9.38)	4 (28.57)	0.052
Altered consciousness	38 (59.38)	2 (14.29)	0.003
ICU admission (%)	25 (39.06)	2 (14.29)	0.120
Anti-nuclear antibody positive (%)	18 (28.13)	1 (7.14)	0.167

anti-NMDAR, anti-N-Methyl-D-Aspartate receptor; MRI, magnetic resonance imaging.

Among cases with anti-NMDAR encephalitis, 6 (9.38%) patients were an anti-SmRNP antibody; 6 (9.38%) patients for an anti-SSA60 antibody; 4 (6.25%) patients for an anti-SSA52 antibody; 2 (3.13%) patients for an anti-SSB antibody. There were none of positive antibodies of an anti-chromosome antibody, an anti-ribosomal protein antibody, an anti-Sm antibody, an anti-SmRNP antibody, an anti-Scl-70 antibody, an anti-myeloperoxidase antibody, anti-protease 3, and an anti-glomerular basement membrane antibody in the anti-NMDAR encephalitis group. Among cases with autoimmune encephalitis with other forms, only 1 patient was ANA positive.

### Comparison of clinical characteristic and clinical outcomes between ANA positive and ANA negative in anti-NMDAR encephalitis

**Figure 1:**
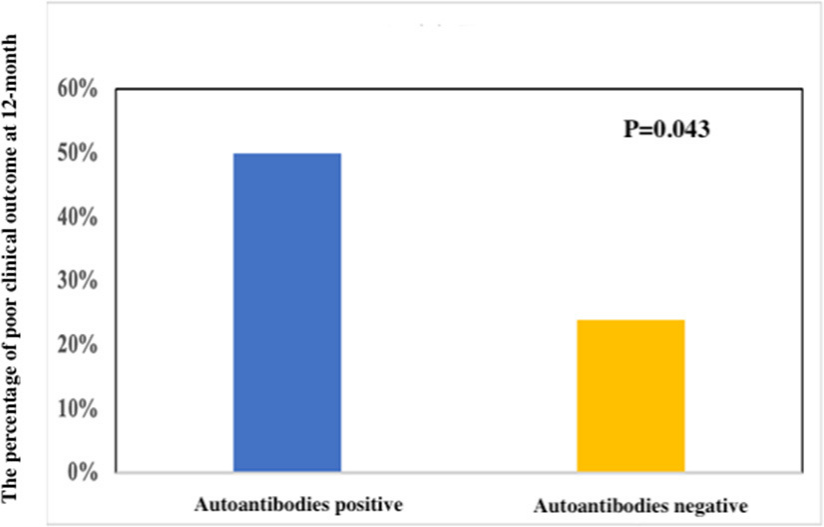
Clinical outcomes between a poor clinical outcome and a good clinical outcome at 12 months in anti-NMDAR encephalitis.

The clinical characteristic and outcomes in anti-NMDAR encephalitis patients with ANA positive (*n* = 18) or ANA negative (*n* = 46) are shown in Table 2. The titers of a positive NMDAR antibody in CSF (*p* = 0.041) and serum (*p* = 0.031) in the ANA-positive group were significantly higher than ANA negative. The patients with ANA positive than those with ANA negative showed lower rates of headache (6 vs. 28; *p* = 0.047) and speech disorder (0 vs. 8; *p* = 0.049). The patients with ANA positive than those with ANA negative were more treated with intravenous immunoglobulin alone. However, age, gender, abnormal MRI, and abnormal EEG were not significantly different. Moreover, there were significant differences of a poor clinical outcome at 12 months (9 vs. 11; *p* = 0.043) (Figure 1).

**Table 2:** The clinical characteristic and clinical outcomes between ANA positive and ANA negative in anti-NMDAR encephalitis.

	**ANA positive (*n* = 18)**	**ANA negative (*n* = 46)**	***P*-value**
Age, years (mean ± SD)	29.67 ± 19.70	27.13 ± 14.27	0.294
Male (%)	10 (55.56)	26 (56.52)	0.994
Abnormal MRI (%)	12 (66.67)	22 (47.83)	0.174
Abnormal electroencephalography (%)	10 (55.56)	16 (34.78)	0.128
**CSF detection**			
CSF NMDAR antibody titers (median, IQR)	1:64 (1:1–1:132)	1: 32 (1:1–1:64)	0.041
Serum NMDAR antibody titers (median, IQR)	1:128 (1:320)	1:64 (1:64–1:128)	0.031
**Treatment (%)**			
Steroids alone	0 (0.00)	8 (17.39)	0.059
Intravenous immunoglobulin alone	4 (22.22)	2 (4.35)	0.048
Combination	14 (77.78)	36 (78.26)	0.996
Rituximab	0 (0.00)	2 (4.35)	1.000
**Prodrome symptoms (%)**			
Fever	8 (44.44)	24 (52.17)	0.578
Headache	6 (33.33)	28 (60.87)	0.047
Dizziness	2 (11.11)	8 (17.39)	0.712
**Clinical symptoms (%)**			
Abnormal behavior	14 (77.78)	30 (65.22)	0.384
Speech disorder	0 (0.00)	8 (17.39)	0.049
Seizures	6 (33.33)	20 (43.48)	0.457
Memory disorder	2 (11.11)	4 (8.70)	1.000
Altered consciousness	12 (66.67)	26 (56.52)	0.457
ICU admission (%)	8 (44.44)	17 (36.97)	0.581
Poor clinical outcome at 12-month (%)	9 (50.00)	11 (23.91)	0.043

### Comparison of clinical characteristics and autoantibodies between poor a clinical outcome and a good clinical outcome in anti-NMDAR encephalitis

The clinical characteristic in patients with anti-NMDAR encephalitis with a good clinical outcome (mRS, 0–2, *n* = 44) or a poor clinical (mRS, 3–6, *n* = 20) outcome at 12 months are shown in Table 3. The patients with a poor outcome than those with a good outcome showed higher rates of altered consciousness (17 vs. 21; *p* = 0.006), ICU admission (18 vs. 7; *p* < 0.001), and positive ANA (18 vs. 7; *p* = 0.043). The titers of a positive NMDAR antibody in CSF (*p* = 0.038) and serum (*p* = 0.012) in the poor clinical outcome group were significantly higher than the good clinical outcome group. However, age, gender, abnormal EEG, and treatments were not significantly different.

**Table 3:** The clinical characteristic and autoantibodies between a poor clinical outcome and a good clinical outcome at 12 months in anti-NMDAR encephalitis.

	**Poor clinical outcome (*n* = 20)**	**Good clinical outcome (*n* = 44)**	***P*-value**
Age, years (mean ± SD)	34.05 ± 15.85	25.02 ± 15.21	0.034
Male (%)	9 (45.00)	27 (61.36)	
Abnormal MRI (%)	14 (70.00)	20 (45.45)	0.068
Abnormal electroencephalography (%)	6 (30.00)	20 (45.45)	0.243
**CSF detection**			
CSF NMDAR antibody titers (median, IQR)	1:64 (1:1–1:132)	1: 32 (1:1–1:64)	0.038
Serum NMDAR antibody titers (median, IQR)	1:128 (1:320)	1:64 (1:1–1:64)	0.012
Steroids alone	0 (0.00)	8 (18.18)	0.049
Intravenous immunoglobulin alone	4 (20.00)	2 (4.55)	0.071
Combination	16 (80.00)	34 (77.27)	0.807
Rituximab	0 (0.00)	2 (4.55)	1.000
**Prodrome symptoms (%)**			
Fever	10 (50.00)	22 (50.00)	1.000
Headache	11 (55.00)	23 (52.27)	0.839
Dizziness	4 (20.00)	6 (13.64)	0.516
**Clinical symptoms (%)**			
Abnormal behavior	14 (70.00)	30 (68.18)	0.884
Speech disorder	2 (10.00)	6 (13.64)	1.000
Seizures	9 (45.00)	17 (38.64)	0.631
Memory disorder	3 (15.00)	3 (6.82)	0.366
Altered consciousness	17 (85.00)	21 (47.73)	0.006
ICU admission (%)	18 (90.00)	7 (15.91)	<0.001

## Discussion

Our results suggested patients with ANA positive had higher titers of a positive NMDAR antibody in CSF and serum. The severity of anti-NMDAR encephalitis was associated with the presence of ANA. Autoantibodies positive may lead to immune dysfunction in the brain by interacting with antibodies directed against neuronal surface antigens, which can trigger a more aggressive autoimmune response against neurons (11). Moreover, the presence of these systemic antibodies drives central nervous system inflammation further worsening the outcome (11). Therefore, anti-NMDAR encephalitis patients with ANA positive may have a worse prognosis. However, the symptoms of headache and speech disorder had an opposite tendency.

One of the main markers of patients with autoimmune diseases was self-antibodies, including RNA, DNA, and other components (7–12). It was useful for the evaluation of autoimmune disease by detecting specific autoantibodies. Thus, it was helpful for diagnosing and differentiating subtypes of autoimmune disease by identifying certain autoantibodies. Moreover, it also was helpful for predicting the progress of clinical manifestations and a prognosis (13, 14). Therefore, we can use systemic autoantibodies to screen and diagnose autoimmune diseases accurately so as to formulate a reasonable, safe, and efficient treatment plan. For patients with anti-NMDAR encephalitis, autoantibodies may be an important biomarker of the diagnosis. In our study, 18 in 64 of patients with anti-NMDAR encephalitis were positive for ANA. The autoimmune disease increases the chance of an additional autoimmune disease, and that patients with autoimmune diseases have a higher rate of positive autoantibodies (15).

A previous study suggested that good outcomes in patients with anti-NMDAR encephalitis were significantly negatively associated with the serum autoantibodies (16). Systemic autoantibodies measured only contained ANAs, ENAs, rheumatoid factors, and ANCAs (16). However, autoantibodies measured in our study included more types of autoantibodies. Recent studies have shown that blood brain barrier (BBB) dysfunction is involved in the main pathophysiological mechanisms of anti-NMDAR encephalitis. Positive autoantibodies were detected in the CSF in patients with neuropsychiatric lupus, which played an important role in the disruption of BBB (18). It was speculated that serum autoantibodies play a role in the damages of neurons by interacting with an anti-NMDAR antibody against neuronal surface antigens, where they can access the brain because of BBB disruption (17, 18).

Our study suggested that higher rates of ICU admission were associated with a poor clinical outcome. Previous studies have shown that a trend for both altered conscious state and ICU admission affects outcomes in anti-NMDAR encephalitis (19, 20). Other studies based on 382 patients also indicated that ICU admission was independent predictors for a poor clinical outcome (1). However, other studies found that, on status, epilepticus was a strong association with patient outcomes, which affected the ICU admission and mechanical ventilation (21). A meta-analysis based on 1,550 patients from 652 articles indicated that infant or older-adult age, ICU admission, an extreme delta brush pattern on EEG, a lack of immunotherapy within 30 days of the onset, and IVIG treatment for 6 months or more were associated with a poor functional outcome (22). This study suggested that therapeutic apheresis alone (5.6-fold increased odds of a good outcome) or first-line treatment options used in combination (2.7-fold increased odds with corticosteroids and IVIG; 2.8-fold increased odds with corticosteroids, IVIG, and therapeutic apheresis) were effective in anti-NMDAR encephalitis (22). Teratoma has been demonstrated to be notably relevant with the occurrence of anti-NMDAR encephalitis. However, our study found that none had teratoma. One Chinese study also reported that no adults underwent tumor resection (22, 23). The test of a potential tumor was dependent of ethnic background, age, and sex.

Our study has some limitations. Firstly, our sample size was relatively small. Thus, it is possible that statistical power was not sufficient to detect small differences, and further investigations are warranted. Secondly, it was a retrospective study, with no standard systematic treatment method (e.g., the selection of treatment, and therapeutic duration for the first- and second-line treatments) and titers of ANA. Thirdly, our study was a preliminary study, which lacks evidence for biological and pathological mechanisms. Fourthly, we did not include patients with anti-NMDAR encephalitis after viral encephalitis or meningitis. Thus, we did not confirm the autoantibody positivity rate in cases of anti-NMDAR encephalitis after viral encephalitis.

Presence of ANA was associated with worse long-term neurological recovery and may need more aggressive immunotherapy. However, this study contains several limitations. Therefore, these findings should be verified by larger studies.

## Data availability statement

The original contributions presented in the study are included in the article/supplementary material, further inquiries can be directed to the corresponding author/s.

## Ethics statement

The studies involving human participants were reviewed and approved by First Affiliated Hospital of Kunming Medical University. The Ethics Committee waived the requirement of written informed consent for participation.

## Author contributions

CL designed the subject and approved the final version of the manuscript. YL, HL, and XZ collected and extracted data of the article. WJ and XC revised the important intelligent content. All authors contributed to the article and approved the submitted version.

## Funding

This study was supported by National Natural Science Foundation of China (82060230), Yunnan young and middle-aged academic and technical training project for high-level talents (202105AC160065), and the Major Science and Technology Special Project of Yunnan Province (202102AA100061).

## Conflict of interest

The authors declare that the research was conducted in the absence of any commercial or financial relationships that could be construed as a potential conflict of interest.

## Publisher's note

All claims expressed in this article are solely those of the authors and do not necessarily represent those of their affiliated organizations, or those of the publisher, the editors and the reviewers. Any product that may be evaluated in this article, or claim that may be made by its manufacturer, is not guaranteed or endorsed by the publisher.
